# How does self-report of anxiety symptoms compare with observer assessments after acquired brain injury?

**DOI:** 10.1192/bjo.2021.765

**Published:** 2021-06-18

**Authors:** Alex Seelochan, Mark Paramlall, Himanshu Tyagi, Rohan Kandasamy, Ida Bakar, Cameron Holloway, Samantha Harding, Anna Gadhvi

**Affiliations:** 1 Northern Ontario School of Medicine; 2 North Bristol NHS Trust, Frenchay Brain Injury Rehabilitation Centre; 3 The National Hospital for Neurology and Neurosurgery, University College London; 4 The National Hospital for Neurology and Neurosurgery, Frenchay Brain Injury Rehabilitation Centre; 5 Frenchay Brain Injury Rehabilitation Centre, North Bristol NHS Trust; 6 Frenchay Brain Injury Rehabilitation Centre; 7 North Bristol NHS Trust

## Abstract

**Aims:**

Comorbid anxiety and mood disorders occur in 30% and 60% of individuals post-ABI (acquired brain injury), respectively (Juengst et al, 2014). The presence of psychiatric symptoms correlate to poorer outcomes in post-stroke rehabilitation, worsened quality of life (QoL), and deficits in memory, attention, and processing speed that persists years following the index event. Despite this, it is unclear whether to what degree anxiety impacts cognition. Furthermore, the literature on this topic is inconsistent when comparing subjective and clinician measurements. This study seeks to ameliorate this gap in literature by analyzing how clinicians’ measures of anxiety and cognitive performance correlate with subjective assessments of patient's own anxiety symptoms.

**Method:**

Individuals with an ABI who were seen in a clinical neuropsychiatry outpatient clinic between 2019 and 2020 completed a GAD-7 (Generalized Anxiety Disorder-7) questionnaire (patient's self-report of the severity of anxiety symptoms) and an observer completed a Neuropsychiatric Inventory Questionnaire (NPIQ) including a subscale for anxiety (NPIQ-A). Participants also underwent a formal cognitive examination with the Montreal Cognitive Assessment (MoCA). A total of 24 ABI patients (depressed ABI and non-depressed ABI) were analyzed for variation, statistical agreement and correlation. Here, total anxiety scores (using GAD-7 scores), anxiety severity (correlating category based on total GAD-7 score) were compared against the objective measures for anxiety (NPI-QA) and cognition (MoCA). In order to standardize MoCA scores, z scores were used in the statistical analysis.

**Result:**

The patient's subjective raw scores of anxiety were statistically significantly different from the corresponding scores from objective observers on Wilcoxon-Rank Sum tests (p < 0.01), however, there was a statistical correlation between GAD (categorized by severity level) and NPI-QA (p = 0.75). Spearman Rank Correlation did show positive, but, statistically insignificant correlation between dyads of these independent variables (including GAD7/NPIQ-A, GAD 7 categorised/NPIQ-A, GAD7/MoCA, GAD 7 categorised/MoCA).

**Conclusion:**

These findings indicate (1) self-reported measures of anxiety (GAD7) in ABI were inconsistent with objective measures of anxiety in this cohort, (2) anxiety measures did not demonstrate significant correlation when compared to objective measures for cognitive function, and (3) ABI patients did not display good insight into the severity of their anxiety symptoms as measured by the GAD7. Further research should focus on utilizing other subjective measurement tools for anxiety and/or clinician evaluation tools with NPIQ-A.

